# EEG Oscillations Reveal Neural Correlates of Evidence Accumulation

**DOI:** 10.3389/fnins.2012.00106

**Published:** 2012-07-17

**Authors:** M. K. van Vugt, P. Simen, L. E. Nystrom, P. Holmes, J. D. Cohen

**Affiliations:** ^1^Department of Artificial Intelligence, University of GroningenGroningen, Netherlands; ^2^Department of Neuroscience, Oberlin CollegeOberlin, OH, USA; ^3^Princeton Neuroscience Institute, Princeton UniversityPrinceton, NJ, USA; ^4^Department of Mechanical and Aerospace Engineering and Program in Applied and Computational Mathematics, Princeton UniversityPrinceton, NJ, USA

**Keywords:** EEG, drift diffusion model, decision making, oscillations

## Abstract

Recent studies have begun to elucidate the neural correlates of evidence accumulation in perceptual decision making, but few of them have used a combined modeling-electrophysiological approach to studying evidence accumulation. We introduce a multivariate approach to EEG analysis with which we can perform a comprehensive search for the neural correlate of dynamics predicted by accumulator models. We show that the dynamics of evidence accumulation are most strongly correlated with ramping of oscillatory power in the 4–9 Hz theta band over the course of a trial, although it also correlates with oscillatory power in other frequency bands. The rate of power decrease in the theta band correlates with individual differences in the parameters of drift diffusion models fitted to individuals’ behavioral data.

## 1 Introduction

Every day we make thousands of decisions, and modeling work has attempted to describe the nature of these decision processes (e.g., Ratcliff, 1978; Usher and McClelland, 2001). With the advent of cognitive neuroscience there has been a growing interest in its neural correlates. Here we introduce a novel approach to studying decision dynamics with human electrophysiology. By using model-predicted decision dynamics as regressors, we perform a comprehensive search for oscillatory features of electroencephalographic (EEG) activity that could reflect evidence accumulation.

There exist two main approaches to analyzing EEG data: looking at the raw potential, averaged time-locked to an event of interest (“event-related potentials”) or looking at periodic activity or oscillations (not necessarily averaged). The presence of oscillations in EEG measurements indicates that neurons in a region have more synchronized synaptic and membrane activity (Wang, 2010). Through being synchronized, oscillations become strong enough in power to be detectable on the scalp. Synchronization is thought to allow groups of neurons to communicate with each other (Womelsdorf et al., 2007; Fries, 2009). Synchronized activity is crucial for plasticity and learning in the brain (STDP; Wang, 2010). The brain also appears to use oscillations in conjunction with spikes to encode specific information. Certain phases of oscillations often show an increased level of spiking relative to their baseline (Fries et al., 2007). A clear example of how the brain makes use of that oscillation-related change in excitability is phase coding, in which the phase of an oscillation at which a neuron fires encodes the spatial location of an animal (O’Keefe and Recce, 1993; Fries et al., 2007).

Without attempting to review the oscillations literature in full, we point out here a few relevant findings of this literature (see Buzsáki, 2006; Wang, 2010, for a more complete review). Probably the most-discussed oscillations are those in the 28–90 Hz gamma band, which have been studied extensively in the context of attention tasks. A prominent finding is that attention increases the amplitude of occipital 28–90 Hz gamma oscillations (e.g., Fries et al., 2007). Yet some studies have shown that oscillations of lower frequency are also important for attention and perception. For example Busch and VanRullen (2010) found that stimuli are better perceived at certain phases of the on-going 4–9 Hz theta oscillation than at other phases. This has led to the idea that so-called sustained attention is not uniformly sustained, but rather has an oscillating quality. Moreover, it suggests that in addition to hippocampal theta oscillations, which have primarily been associated with memory (e.g., Kahana et al., 2001) and spatial navigation (e.g., O’Keefe and Burgess, 1999), there exist cortical theta oscillations that are relevant to, among other things, perception.

In fact, it has also been suggested that cortical theta oscillations are crucial for the coordination of multiple sources of activity at decision points (Womelsdorf et al., 2010), and for combining various pieces of evidence (van Vugt et al., in press). Theta oscillations have also been found to covary with decision certainty (Jacobs et al., 2006) and prediction errors in decision making (Cavanagh et al., 2010). This suggests that theta oscillations could have a fundamental role in perceptual decision making and specifically in the accumulation of evidence. Nevertheless, other sources suggest that evidence accumulation is correlated with higher frequency oscillations in the beta and gamma bands (e.g., Donner et al., 2009).

The aim of this study is therefore to use a data-driven approach to find oscillatory correlates of evidence accumulation. To be able to do so, we need precise predictions for the dynamics of evidence accumulation provided by mathematical models. Probably the most-discussed model for evidence accumulation is the Drift Diffusion Model (DDM; Ratcliff, 1978). This model posits that to make a decision, a person accumulates information until it reaches a threshold, at which time they make the response that corresponds to that threshold. Their response times (RTs) can be predicted by adding a fixed non-decision time to the time it takes to reach the threshold to account for sensory and motor latencies. The speed with which one accumulates evidence on average is referred to as the “drift rate” of the accumulation process. The height of the decision threshold reflects response caution. This model, and variants of it, is capable of explaining complete RT distributions, not just average RTs like most other models of cognition (Ratcliff and Smith, 2004).

In this study we examine what frequency bands of brain oscillations best reflect evidence accumulation as predicted by accumulator models. We also test whether the dynamics of the thus-selected oscillations covary with individual differences in DDM parameters estimated on the basis of participants’ behavioral data. This work not only furthers our understanding of human decision making, but may eventually allow us to distinguish different implementations of the DDM that cannot be disentangled based on behavioral data alone (Ditterich, 2010).

## 2 Materials and Methods

### 2.1 Task

Participants performed a perceptual decision making task in which they judged the direction of motion (left or right) of a display of randomly moving dots of which a percentage moved to the left or the right. These random dot kinematograms were similar to those used in a series of psychophysical and decision making experiments with monkeys as participants (e.g., Britten et al., 1992; Shadlen and Newsome, 2001; Gold and Shadlen, 2003). Stimuli consisted of an aperture of ~7.6 cm diameter viewed from ~100 cm (~4° visual angle) in which white dots (2 × 2 pixels) moved on a black background. A subset of dots moved coherently either to the left or to the right on each trial, whereas the remainder of dots jumped randomly from frame to frame. Motion coherence was defined as the percentage of coherently moving dots. Dot density was 17 dots/square degree, selected such that individual dots could not easily be tracked. Tracking was further discouraged by using three interleaved sets of dots of equal size, each of which was used in every three successive video frames. Therefore each set of dots returned only after three frames, with a random displacement. The speed of the dots was ~7°/s. Participants indicated their responses by pressing the “Z” key with their left index finger for left-ward motion or the “M” key with their right index finger for right-ward motion.

Participants also performed a control task in which they did not need to integrate motion evidence (non-integration condition). In this condition, each trial started with entirely random (0% coherence) dot-motion, followed by an arrow indicating the direction to which a participant should respond. The arrow onset time was calibrated (based on RTs in previous blocks of the non-integration condition) such that the dot-motion-viewing times in these trials mirrored the response time distribution of the dots trials. This was done by taking the RT distributions from previous blocks, and subtracting from that the average time required for pressing a button in response to a stimulus (“signal detection trials”).

The experiment presentation code was written in PsychToolbox (Brainard, 1997). Dots were presented with PsychToolbox extensions written by J. I. Gold^1^.

### 2.2 Participants

Twenty-three participants (12 female; 21 right-handed; mean age 25; range 18–38) participated in our experiment in exchange for payment. The experiment was approved by the Institutional Review Board of Princeton University. Participants engaged in three separate hour-long training sessions in which they became familiar with the task. At the beginning of these training sessions, performance on a psychometric block (with fixed viewing times of 1000 ms and five different coherence levels) was used to determine the coherences at which they performed at ~70 and 90% correct. These coherence levels were used for the remainder of the session, and the coherences from the last training session were used for the two EEG sessions.

### 2.3 Recording methods

We recorded EEG data from 128 channels using Neuroscan EEG caps with a Sensorium EPA-6 amplifier. Data were digitized at 1000 Hz and band-pass filtered from 0.02 to 300 Hz; all impedances <30 kΩ. Acquisition was controlled by Cogniscan software. All data were referenced to the left mastoid and off-line rereferenced to an average reference after automatic bad-channel removal (Friederici et al., 2000; Hestvik et al., 2007). We wavelet-transformed the data in six standard frequency bands (delta (2–4 Hz), theta (4–9 Hz), alpha (9–14 Hz), beta (14–28 Hz), low gamma (28–48 Hz), and high gamma (48–90 Hz); van Vugt et al., 2010) using six-cycle Morlet wavelets. Morlet wavelets have an optimal trade-off between temporal- and frequency resolution for EEG data (van Vugt et al., 2007) and six cycles are often used for the analysis of EEG data (e.g., Caplan et al., 2001).

### 2.4 General linear model for EEG

To find neural correlates of the dynamics of decision making in EEG data, we developed a General Linear Model (GLM) method, in which we correlated the predicted accumulator dynamics with the EEG time series. GLMs are a type of regression that is generally used with functional magnetic resonance imaging (fMRI) to find voxels that display a hypothesized pattern of activation, such as “high” when an object is presented and “low” when a scrambled object is presented. Here we used a similar technique with EEG data, to detect electrodes that display a pattern of activation that is predicted by the DDM.

**Figure 1:**
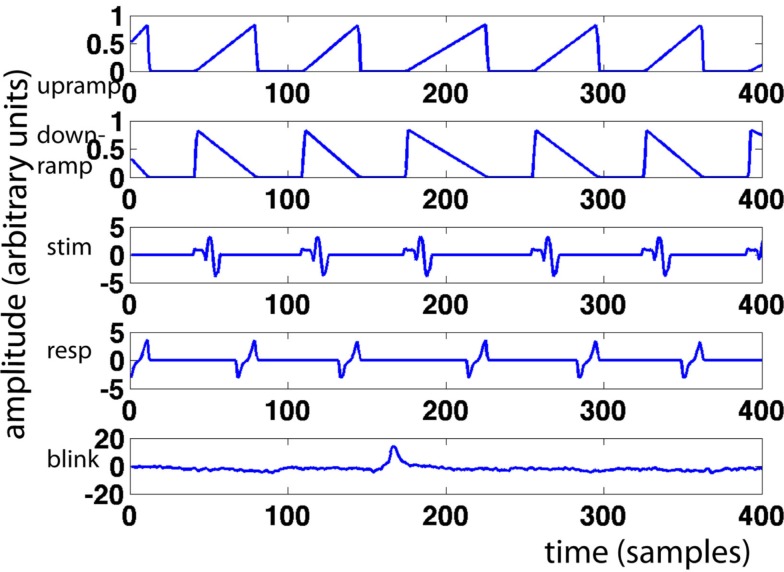
**Sample regressors**. Pictured are, from top to bottom: upramp, downramp, stimulus regressor, response regressor, and eyeblink regressor. *X*-axis represents time in samples, *y*-axis regressor amplitude in arbitrary units.

For every trial, we modeled a ramp of activity starting at stimulus onset and ending at the response. This “upramp” had a height of one, and its slope was constrained by the response time for that trial (see Figure 1 for examples). We compared the correlations of the ramp regressors to those of regressors reflecting the alternative hypotheses of neural activity that is “on” during the trial (“boxcar”) and of neural activity that reflects a transient initial response slowly decreasing over the trial duration (“downramp”). Evidence accumulation activity extracted with the upramp regressor should look clearly different from these alternative hypotheses. We did not employ separate regressors for left- and right-ward dot-motion. In other work (van Vugt et al., in revision), we have similarly detected lateralized readiness potentials, but these depart from baseline much later than the theta band activity discussed below, and we believe they are primarily motor (response) related.

We created a set of parallel upramps, downramps, and boxcars for the arrow control task, which had the same height as the dots ramps (i.e., a unit height), and also started at dots onset and ended at dots offset. Note, however, that in that case the arrows appeared somewhere in the middle of the interval between dots onset and offset, and the response followed fairly promptly thereafter.

**Figure 2:**
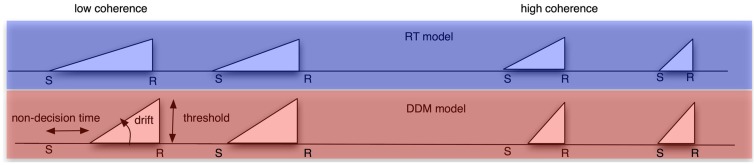
**Schematic of the different models we used to create regressors from**. Top row: RT model. Bottom row: DDM model. Left column: low-coherence condition. Right column: high-coherence condition. In the RT model, the ramp always starts at stimulus onset and ends always at the response, and it always has a height of one. It therefore has a different length for the slower and faster trials within a coherence condition. Conversely, the DDM model has a fixed shape for all trials within the low-coherence condition, and another shape for all trials within the high-coherence condition. This shape is determined by three DDM parameters: non-decision time (which determines ramp onset), decision threshold (which determines ramp height) and drift rate (which determines the slope of the ramp).

We compared the fits of these regressors to an alternative model that did not take trial-by-trial variation in response time (RT) into account. In that model, we created regressors with a slope and height modulated by the individual’s drift rate and threshold, respectively. In addition, the slope did not depart from baseline until T_0_ (non-decision time) milliseconds after stimulus onset. We created one such regressor for the low-coherence condition, and a second for the high-coherence condition. We inserted in each trial the respective regressor shape. Importantly, these regressors did not covary with individual trial RTs (see Figure 2). As a result, the DDM model only has five parameters (low- and high-coherence drift rates, low- and high-coherence non-decision times, and decision thresholds), whereas the RT model has as many parameters as there are trials (namely, the RT for every trial). The DDM parameters were obtained by fitting the pure DDM (i.e., a DDM without variability in starting point, non-decision time, and drift rates) to each participant’s behavioral data with the DMA toolbox (VandeKerckhove and Tuerlinckx, 2007, 2008).

**Figure 3:**
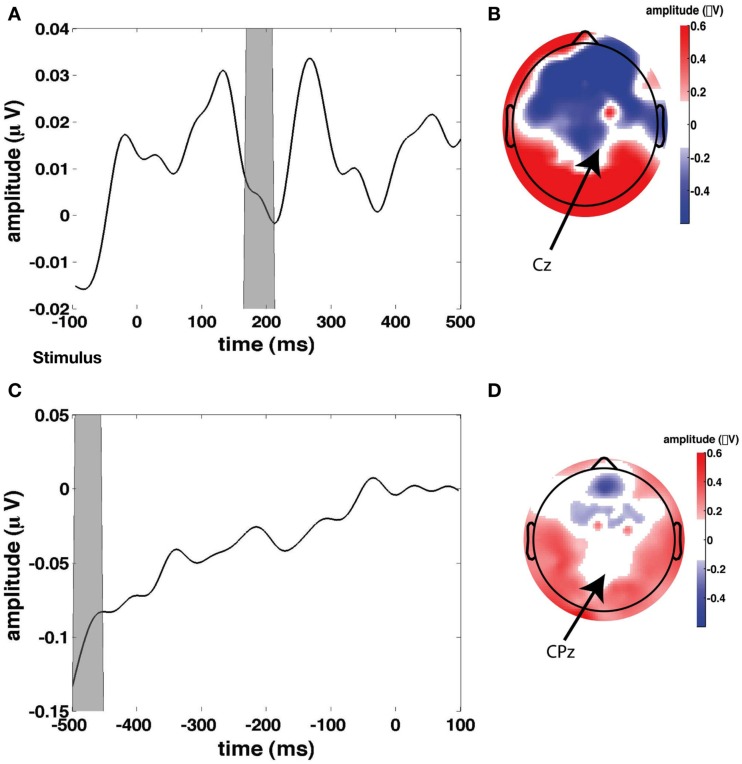
**Stimulus and response-locked event-related potentials (ERPs) used for creating the stimulus and response regressors**. **(A)** Grand average stimulus-locked ERP (i.e., average across all channels). **(B)** Topographical plot of ERP amplitude in the gray time window in the time course in **(A)** which represents the first stimulus-evoked peak. **(C)** Grand average response-locked ERP. **(D)** Topographical plot of ERP amplitude in the gray time window in the time course in **(C)** which represents the maximum response-related peak.

In addition to these regressors of interest, we created a set of nuisance regressors that modeled transient neural responses to stimulus onset and button press, as well as eye activity. These nuisance regressors are used to remove those sources of variance from the EEG signal, such that only the signal of interest remained. To determine the transient response to a stimulus, we first looked at the grand average of stimulus-related EEG activity (Figure 3A), from which we chose a time window to plot a topographical distribution (Figure 3B). Although this topography does not exhibit a single clear maximum, we chose to use electrode Cz to compute for every participant individually the stimulus-related average. We then inserted this average waveform (from 0 to 300 ms post-stimulus) in the regressor at any time point where a stimulus was presented (“stimulus regressor”).

Similarly, we examined the grand average response-locked ERP (Figure 3C) to define a time window for which to compute a topographical plot (Figure 3D) which was then used to define a response-related electrode. We chose CPz (Cz is slightly more anterior than CPz). We then used the average response-locked waveform from −200 to 0 ms in CPz to model the transient neural response to a button press (“response regressor”). See Figure 3 for an illustration of the locations of Cz and CPz. Response-related ERP peaks typically exhibit their maximum more posteriorly than stimulus-related ERP peaks. CPz and Cz are two central electrodes that show peak responses to stimulus presentation and button presses, respectively. The eye blink regressor was created from the activity of the eye channel. We focused exclusively on eye blinks and not on horizontal eye movements because we only collected eye movements from a single eye electrode placed underneath the left eye. We set the eye blink regressor to zero outside the eye blink episodes detected with an amplitude threshold to ensure that no random fluctuations in the eye channel could distort our results.

A major problem in GLM analyses of EEG data is the poor signal-to-noise ratio (SNR). To improve the SNR we created features (independent variables in the regression) that only consisted of the trials themselves. This thus excluded the inter-trial time in which participants may have moved or been engaged in unspecified cognitive processes such as contemplating their lunch). We padded the trials with 300 ms before the stimulus and after the response. The reason for including this extra-trial time is that the neural activity of interest should display the hypothesized ramping behavior during the trial, but should also be relatively quiet outside this period, since at that time no evidence is being accumulated, and participants’ attention will not yet have wandered very far away just after the trials. Moreover, not including this extra-trial period will make the upramp and downramp regressors identical up to an inversion, and this causes problems for the analysis. We can exploit the additional variance provided by this baseline to find our signal of interest in the EEG data. We then appended all these padded trials into a feature vector. The features were created both from the raw EEG time series, and wavelet-convolved signals in the delta (2–4 Hz), theta (4–9 Hz), alpha (9–14 Hz), beta (14–28 Hz), low gamma (28–48 Hz), and high gamma (48–90 Hz) ranges. After construction, we downsampled these features to 50 Hz, and *z*-transformed them to put them on the same scale across participants (van Vugt et al., 2010). Downsampling was done to reduce memory load for the computations.

We ran the GLM in two steps. In the first step we modeled all the nuisance regressors. The regressors of interest (ramps) were then modeled on the residuals of this first regression, which ensured that the nuisance regressors could not influence the fits for the regressors of interest. In addition to computing the regression coefficients for each feature, we also computed the (square root of the) variance explained by correlating the feature with the fitted regressors as a measure of goodness of fit (Tabachnick and Fidell, 2005).

### 2.5 Multivariate group analysis

**Figure 4:**
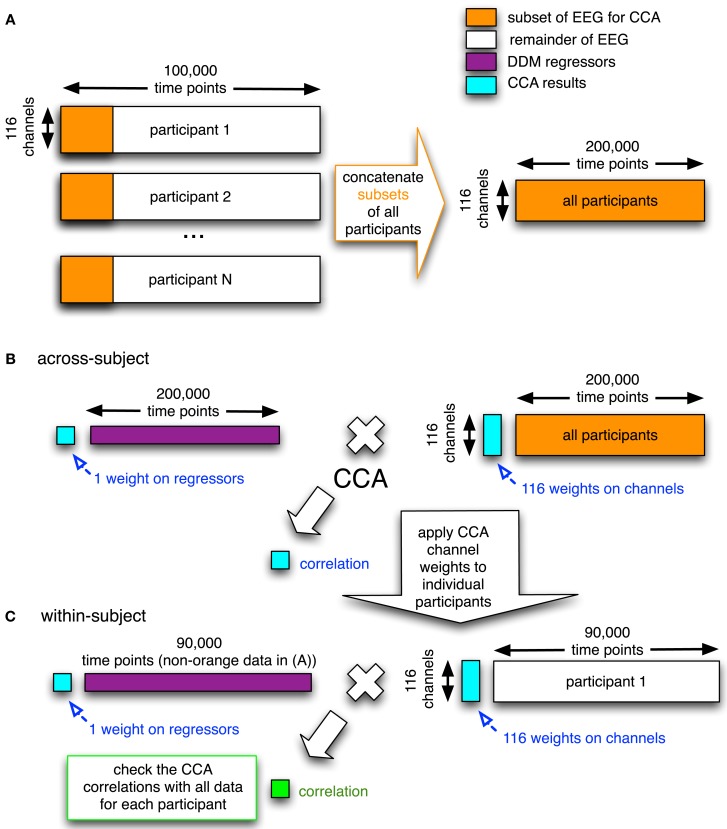
**Graphical overview of the CCA/GLM method we developed**. In the first step **(A)**, the electrodes-by-time matrices are concatenated in the time dimension for all participants, where only a subset of the data of each participant is used (orange rectangles). This concatenated matrix with EEG data is then used together with the corresponding concatenation of regressors (purple; the DDM-inspired model time series) in the second step. **(B)** In this second step, the electrode-by-time matrix that contains data from all participants is correlated with the corresponding time course of the regressor (e.g., upramp) using CCA. This yields a correlation value, a weight on the regressor, and a set of weights on the electrodes (all in cyan). In the third step **(C)**, the weight map on the electrodes is applied to the remainder of every participants’ EEG data (white) and the correlation (green) of this weighted EEG data to the regressor (purple) is compared to the correlation based on the group data in the random effects analysis (cyan). This whole procedure is done for the raw EEG data and separately for each frequency band (cf. Figure 9 below). The number of time points indicated above the matrices are just an indication.

To combine across participants, we included all participants’ data into a single canonical correlation analysis (CCA; Calhoun et al., 2009). In general, CCA is a multivariate technique to find correlated components between two datasets. When given two matrices (e.g., a set of regressors and a set of time courses from electrodes), it finds a set of weights on these two matrices such that they are maximally correlated. The CCA method we used (see Figure 4) was designed to do a group analysis across all of the participants.

There is no clear agreement in the literature on how to combine data across participants when using decomposition methods such as ICA and CCA (Calhoun et al., 2001). One approach is to perform the decomposition for each participant separately and then sort the resulting components. A problem with that method is that it is unclear how this sorting should be done reliably because the decomposition may have resulted in somewhat different components for every participant. Another approach is to concatenate all participants’ data and to perform the decomposition on the resulting group data. The advantage of this method is that there is only a single set of weights on electrodes across all participants (i.e., a common spatial structure), while each participant has their own time course. It therefore becomes very easy to compare results across participants. A disadvantage of this method is that it forces components to have a similar spatial distribution across participants (Calhoun and Eichele, 2010). However, the added benefit of increased reliability derived from not having to aggregate potentially disparate components outweighed this limitation.

In this study we therefore decided to use temporal concatenation, such that we still had 116 features (corresponding to one feature for every EEG channel) but had hundreds of thousands of time points (see Figure 4A). The CCA then resulted in a single set of canonical correlation weights on electrodes across participants. We had sufficiently many time points to run into memory problems. To overcome these, we took a representative subset of each participants’ data (i.e., six 4-min blocks from each of the different conditions). We checked that including only a subset of each participants’ data in the group decomposition is a reasonable approach in Figure 10 (see Results for more details). We computed a Bayes Factor to check whether indeed there was no strong evidence for a difference between the subset of the data and the full data by using the Bayes Factor calculator^2^ (Rouder et al., 2009).

The CCA produced a single set of electrode weights across all participants for each regressor (Figure 4B). These electrode weights can take either sign, a negative correlation with an upramp implying that power decreases as time progresses.

### 2.6 Random effects analysis

We developed a random effects analysis as a complementary means to assess the significance of the various correlations between regressors and EEG data. To this, we made use of the fact that we had only used a subset of each participants’ data to run the CCA. We used the remaining data to compute for each participant separately a correlation between the EEG data to which the CCA-derived electrode weights were applied, and the corresponding regressors. We then Fisher-transformed these correlations and compared them with *t*-tests. As such, we could for example assess whether the correlation between EEG and the dots time course was larger than the correlation between EEG and the arrows time course. Because this involved many *t*-tests, we applied a False Discovery Rate procedure (Benjamini and Hochberg, 1995). A False Discovery Rate of 0.001, which is the level we used, indicates that on average 1 in 1000 of the significant effects we find is a false positive.

### 2.7 Permutation analysis

Since it is possible that the results we obtained were due to random correlations between the EEG data and the regressors, we performed an additional permutation analysis to assess what the levels of canonical correlation would be for random data. We created random data by shuffling the ramps in time for random amounts and repeated the canonical correlation analysis with these regressors. We did this for 1000 iterations. We then compared the correlations obtained from the empirical data to those obtained from the random data, and computed the probability that our empirical data were derived from these random distributions.

## 3 Results

### 3.1 Behavioral data

**Figure 5:**
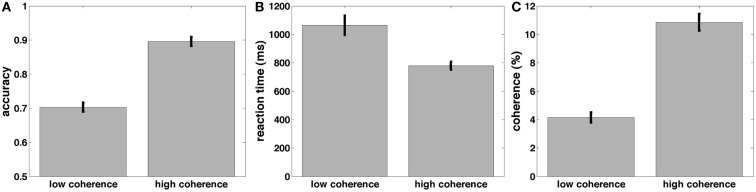
**Mean accuracy (A), response time (B), and coherences (C) across subjects for the low and high-coherence (difficult and easy) task conditions**.

Before turning to the electrophysiological results, we discuss our behavioral data. Participants were engaged in a random dot-motion discrimination paradigm, where the level of motion coherence was set such that they performed at ~70 and 90% correct (Figure 5C). Accuracy was significantly higher [*t*(22) = 21.6, *p* < 0.001, Figure 5A] and RT was significantly faster [*t*(22) = 5.7, *p* < 0.001, Figure 5B] in the 90% correct condition. The two coherence levels used to create the 70 and 90% correct conditions were also significantly different from each other [*t*(22) = 17.3, *p* < 0.001].

These results are consistent with a DDM parametrization in which thresholds are approximately constant, starting points are approximately midway between thresholds, and the drift rate is high for the high-coherence condition, and low for the low-coherence condition. This was confirmed by fitting a DDM to the behavioral data (Table 1). The mean (sem) Maximum Likelihood of these fits across subjects was 3466 (167) and the mean BIC (Bayesian Information Criterion) was 3508 (167). The fits showed that indeed the drift rate was higher for the high-coherence compared to the low-coherence condition. The drift rate was even higher for the arrows non-integration control condition, where evidence was so abundantly clear that participants barely needed to integrate information.

**Table 1:** **Mean (sem) DDM parameters for best fitting model to data from Experiment 1, separately for low and high-coherence trials (integration conditions), and arrows trials (non-integration condition)**.

Condition	Drift	Decision threshold	Non-decision time	Starting point
Low-coherence	0.057 (0.004)	0.151 (0.007)	0.439 (0.011)	0.077 (0.004)
High-coherence	0.167 (0.008)	0.151 (0.007)	0.408 (0.014)	0.078 (0.005)
Arrows control	0.784 (0.069)	0.210 (0.036)	0.219 (0.009)	0.094 (0.027)

*The threshold was held constant between the low and high-coherence conditions. The fits adhere to the Ratcliff convention where the non-biased starting point is half the decision threshold value*.

### 3.2 Electrophysiological data

**Figure 6:**
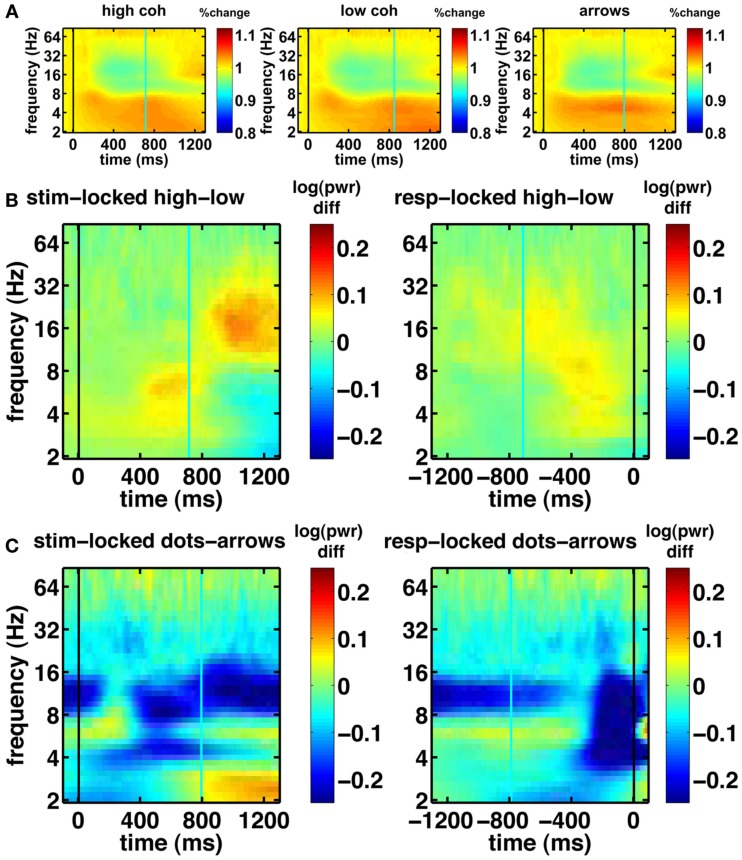
**Basic spectrograms for electrode Cz (a representative electrode)**. Black line indicates the event to which the data are aligned (onset of dot-motion for the stimulus-locked graphs and the response for the response-locked graphs). Cyan line indicates the average response time and stimulus onset time, respectively. **(A)** stimulus-locked spectrograms for the high-coherence, low-coherence, and non-integration condition. There is a gradual decrease in oscillatory power over the course of the trial. **(B)** Difference spectrograms comparing low- and high-coherence conditions. Left column: stimulus-locked. Right column: response-locked. **(C)** Difference spectrograms indicating the contrast between integration and non-integration conditions. Left column: stimulus-locked. Right column: response-locked.

Before turning to our novel model-based EEG analyses, we first examine the basic EEG data. We looked at all electrodes and picked a few representative samples of standard electrodes that are typically shown in EEG studies. Figure 6 shows the basic characteristics of our EEG data. The spectrograms of oscillatory power for central electrode Cz show task modulation and a decrease in 4–9 Hz theta power over the course of the trial (Figure 6A). Figure 6B shows the effect of motion coherence (i.e., task difficulty), where trials in the easy high-coherence condition tend to show higher theta power near the response, compared to trials in the more difficult low-coherence condition. There also seems to be a difference in 14–28 Hz beta power occurring after the mean response time. This may reflect motor activity, which occurs later for the difficult compared to the easy trials, because the difficult trials have longer response times. Figure 6C shows the difference between the integration (dot-motion) and non-integration (arrows) conditions. Across the whole task, the non-integration condition is associated with higher 9–14 Hz alpha power than the integration condition. Furthermore, a decrease in theta is visible between stimulus and response, where the response is associated with lower theta.

**Figure 7:**
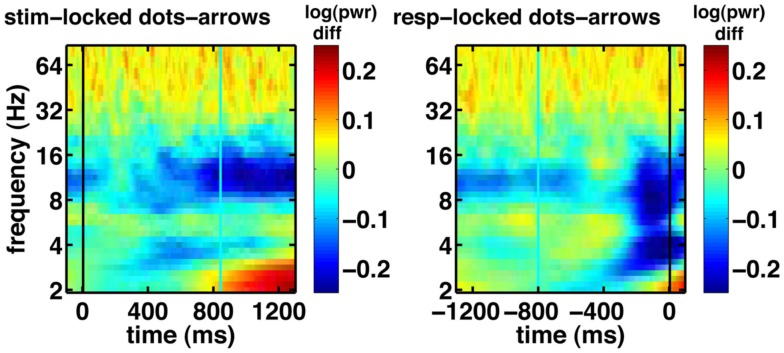
**Basic electrophysiological data for electrode FPz**. Black line indicates the event to which the data are aligned (onset of dot-motion for the stimulus-locked graphs and the response for the response-locked graphs). Cyan line indicates the average response time and stimulus onset time, respectively. These are difference spectrograms contrasting the integration and non-integration conditions. Left column: stimulus-locked. Right column: response-locked.

Another notable effect that can be observed in the raw data is increased gamma oscillatory power for the integration condition, compared to the non-integration condition (shown for the frontal electrode FPz in Figure 7). This difference appears to be fairly constant across the whole task period. On the basis of these spectrograms, we expect evidence accumulation dynamics in the form of ramps (Figure 1) to be primarily associated with low-frequency oscillations as shown in Figure 6A.

### 3.3 GLM method checks

**Figure 8:**
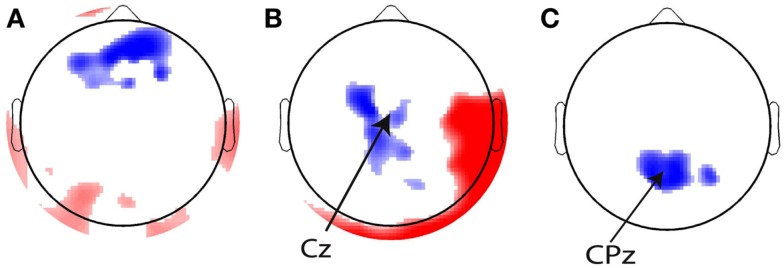
**Topographical overviews of the 10% most significant electrodes for the (A) eye blink, (B) stimulus, and (C) response regressors**. Red and blue reflect positive and negative regression weights, respectively, and the intensity of the shading indicates the magnitude of the regression weights. The most significant electrodes are in the locations from which the regressors were generated [indicated with arrows in **(B,C)**].

Before running the CCA on the ramp regressors, we verified our method by plotting the weights for the eye blink, stimulus, and response regressors on a topographical plot. If the GLM method works correctly, we expect the highest regression coefficients on the electrodes that were used to generate the relevant regressor. That is, for the eyeblink regressor, we expect the largest weights on the frontal electrodes. For the stimulus regressor that was generated based on Cz, the maximum correlation should occur with this electrode, and the response regressor should maximally correlate with CPz. Figure 8 shows that this was indeed the case.

Having established that the GLM, which predicts the time series of a single electrode by a linear combination of regressors, is a viable method to analyze EEG data, we used the multivariate CCA method to search for the hypothesized ramp dynamics in our data. The advantage of CCA is that rather than focusing on a single electrode at a time, it allows linear combinations of channels to predict linear combinations of regressors. It is therefore much more flexible. Yet, because it is more difficult to interpret linear combinations of regressors than single regressors, we restricted our attention to single regressors. Nevertheless, preliminary observations indicate that ~40% larger canonical correlations can be obtained by allowing linear combinations of regressors. Linear combinations of regressors could for example create boxcars from a roughly equal weighting of upramp and downramp regressors, in a completely data-driven manner. Future work should further explore this option.

We performed a CCA between the regressors and the EEG time series for every channel. We did this analysis separately for every frequency band (2–4 Hz delta; 4–9 Hz theta; 9–14 Hz alpha; 14–28 Hz beta; 28–48 Hz low gamma; 48–90 Hz high gamma) because we sought to make inferences about which band shows most evidence of ramping activity.

**Figure 9:**
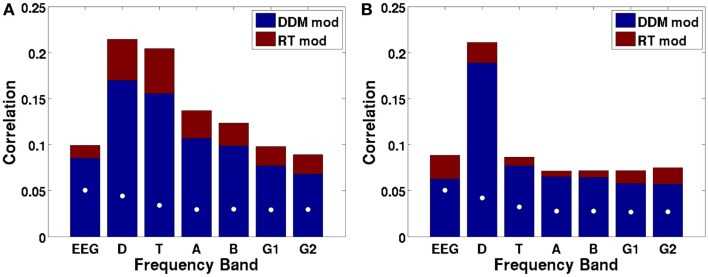
**Canonical correlations as a function of frequency, with the DDM-modulated model in blue and additional correlation achieved by the RT-modulated model in red, shown separately for the ramp regressor of (A) dots (integration condition) and (B) arrows (non-integration condition)**. White dots indicate the 97.5th percentile of the distribution of canonical correlations expected based on random regressors. Letters indicate frequency bands: EEG = raw EEG, D = 2–4 Hz delta, T = 4–9 Hz theta, A = 9–14 Hz alpha, B = 14–28 Hz beta, G1 = 28–48 Hz low gamma, and G2 = 48–90 Hz high gamma.

Figure 9A shows the canonical correlation of the upramp with EEG data in every frequency band, as well as for non-wavelet-transformed (plain) EEG. The correlation between hypothesized ramping dynamics and EEG activity is largest in the 2–4 Hz delta and 4–9 Hz theta bands. Note that all correlations given by CCA are constrained to be positive, and that any negative relations between regressors and EEG will be reflected in a negative sign of the weights on the electrodes. We did a random effects analysis as a follow-up, in which we applied the CCA-derived electrode weights to each participants’ left-out EEG data (only a subset of each participants’ data was used for CCA due to memory constraints) and computed the correlation between these data and the regressors. We then assessed whether the Fisher-transformed individual-subject correlation values were significantly different from zero. We found that this was the case for all frequencies, except for the high gamma band [all *t*-values up to low gamma >6, which reflects *p*-values smaller than 0.0005, which is the *p*-value level corresponding to a False Discovery Rate of 0.001].

We also assessed whether the different frequency bands reflected evidence accumulation or rather a more general dot-motion-induced ramping process by repeating the same analysis for the non-integration condition. Figure 9B shows that the canonical correlate in the theta band is specific to the integration (dots) condition, whereas the canonical correlate in the delta band is also fairly high for the non-integration (arrows) condition. Indeed, comparing the distributions of individual-participant correlation values between the dots and arrows conditions for the delta band indicates that those are not significantly different when using a False Discovery Rate of 0.001 [*t*(22) = 2.98, *p* = 0.0069]. This means that in the non-integration condition, delta oscillatory power increases (or decreases-depending on the sign of the canonical correlation weights) over the course of a trial from dots onset until response, while according to our theory the participant only starts to accumulate evidence (and does so quite rapidly) once the arrow stimulus appears on the screen at a later point.

Theta oscillations show a dramatic drop in (canonical) correlation value in the non-integration condition, as would be expected from a neural correlate of evidence accumulation, because in the non-integration condition virtually no evidence has to be accumulated, and evidence accumulation only starts when the arrows appear on the screen. Indeed, there is a significant difference between the correlations of theta power with the upramp for dots versus arrows conditions [*t*(22) = 11.6, *p* < 0.001]. This suggests that theta oscillations are a more likely candidate for a neural correlate of evidence accumulation than delta oscillations because only the theta oscillations are specific to the integration condition.

We further tested whether the correlations we obtained could be due to a better match between temporal structure of the EEG data in the delta and theta frequency bands and the structure of the regressor than with EEG data in other frequency bands. In other words, we tested the alternative hypothesis that any random sequence of ramps would produce the correlations we observed, with the highest correlations in the delta and theta bands. To that end, we performed a permutation test. We created a set of 1000 regressors in which we randomly moved the ramps around across time, and redid the CCA. The white dots in Figure 9 indicate the 97.5th percentile of the distribution of correlations that would be expected based on random regressors. These random correlations are clearly below the observed correlations. Moreover, all of the observed canonical correlations based on the empirical data are larger than the canonical correlations based on random data, so the probability that the empirical canonical correlations are obtained from random data is <0.001.

**Figure 10:**
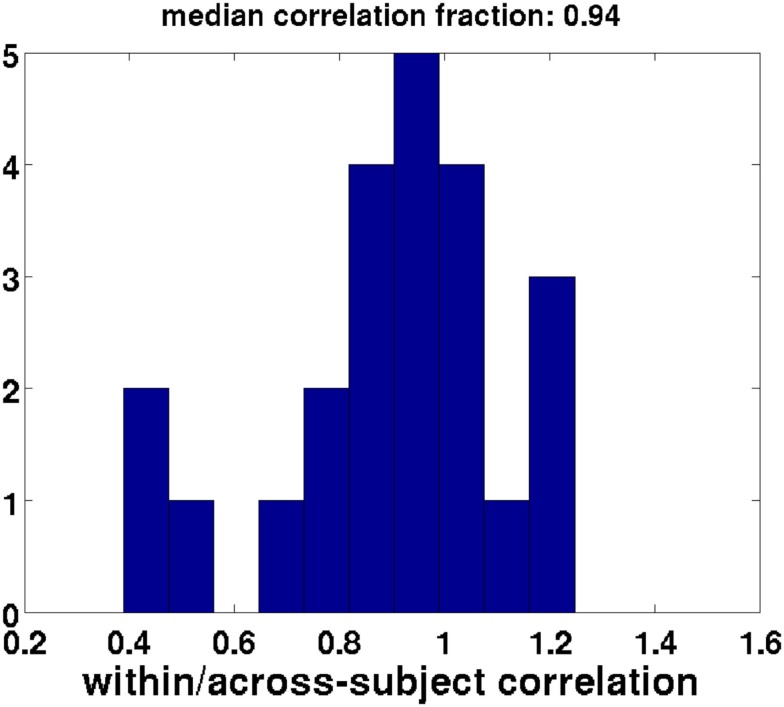
**Validation of the subset method: within-subject correlation of weighted regressors with EEG data divided by the across-subject canonical correlates**. Perfect validity of the subset method would yield a fraction of one (within-subject correlations equal to across-subject canonical correlation). Each datapoint used to create this histogram reflects a single participant. This distribution has a mean that is not different from one.

We further asked whether our CCA, which was based on only a subset of each participant’s data (see Methods), is a good representation of the participant’s data. To this end we compared the correlation value of the canonical correlate of interest with the correlations between the regressor and the weighted set of electrodes for the entire time course within each subject (i.e., using the complete data for each subject). If the CCA decomposition were the same for every participant, then the canonical correlation of the across-subject data would be identical to the within-subject correlation based on the same weights. Figure 10 shows that the within-subject correlations based on a subject’s complete data, weighted by the coefficients obtained from the CCA, have generally similar values to the across-subject CCA based on a subset of a subject’s data [one-sample *t*-test comparing mean proportion between the within and across-subject correlations to one: *t*(22) = 2.0, *p* < 0.1, Bayes factor for the alternative hypothesis of a mean correlation different from one versus the null hypothesis of a mean correlation equal to one: 1.02, indicating there is also little evidence for the null hypothesis].

We then wondered to what extent EEG activity would show correlation with alternative patterns of activity. Rather than upramps, there could also be neural correlates of downward ramps, which start at a high level and then return to baseline by the time of the response. Note that such a downramp is clearly different from a negative correlation of EEG with an upramp: the downramp starts at a high level and then drops down to baseline by the end of the trial, while the negative upramp starts at baseline and then goes down to a level of −1 at the end of the trial. The inclusion of between-trial baseline data allows us to distinguish between negative weights on upramps and (positive weights on) downramps. Finally, there could be patterns of neural activity that turn on at the start of the trial, and turn off at the end that reflects task engagement. We modeled this with a boxcar between stimulus onset and response.

Our results show that potential accumulators (upramps) are more consistent with the EEG data than these alternative hypothesis. Both downramp and boxcar regressors showed much lower correlations [in the theta band the canonical correlations are 0.11 for the boxcar, 0.076 for downramp dots, and 0.15 for downramp arrows]. This was corroborated by a random effects analysis, which showed that for all but the high gamma band, the upramp had a significantly higher correlation than the downramp [all *t*s > 6.04, *p* < 0.001].

We then asked to what extent the DDM, free from trial-to-trial variations in RT, could predict our EEG data. To do this, we compared the canonical correlations for a regressor that was ramping up or down exactly in accord with RT to that of a regressor that was more stereotyped, having a fixed length (time-locked to the response) but modulated by an individual’s DDM parameters as obtained from fitting the DDM to the participant’s behavioral data. Regressor height was modulated by the threshold parameter; its slope by the drift parameter and ramp onset was delayed by the participants’ non-decision time (see Figure 2). Because the DDM-modulated regressor is not yoked to RT, it fails to capture the stochastic noise in RT. Although, as would be expected, the canonical correlations are uniformly higher for the RT-based regressor than for the DDM-modulated regressor, it is remarkable that the DDM still explains a large fraction (0.58–0.73) of the variance that the RT-yoked regressor can (Figure 9, red boxes labeled RT mod). In other words, the model is able to account for a large portion of the neural variance in ramp-like behavior.

**Figure 11:**
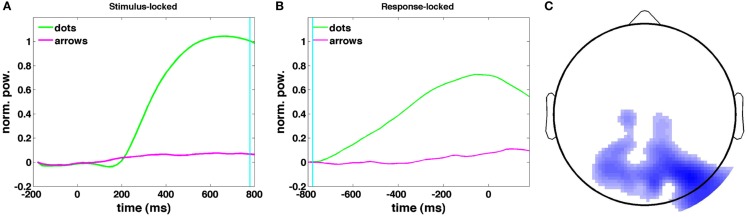
**Stimulus-locked (A) and response-locked (B) time courses of the canonical correlate in the 4–9 Hz theta band, correlated with the dots upramp, cyan line indicates RT in (A) and dots onset in (B)**. **(C)** Dots upramp topography (red indicates a positive correlation between oscillatory power and the regressor, blue a negative correlation). The shade of the color indicates the magnitude of the correlation. Note that there are no positive correlations for the dots upramp.

Figures 11A,B show the time courses of the canonical correlate in the theta band: the frequency band that shows the greatest difference in upramp weights between dots and arrows (Figure 9). The time course of the upramp regressor is much more peaked for the integration condition (green) than for the non-integration condition (arrow trials; magenta). In the stimulus-locked average, the dots upramp time course departs significantly from baseline around 240 ms [one-tailed *t*-test with a *p* < 0.01 significance level], whereas the arrows upramp time course does not depart significantly from baseline until 400 ms post-stimulus. From 260 ms post-stimulus, the dots upramp and arrows upramp are significantly different. Similarly, in the response-locked time courses, the dots upramp differs from baseline from −580 ms, but the arrows upramp not until −380 ms. The two time courses start to differ significantly at −520 ms. The smaller amplitude of the arrows upramp is what we expected based on the lower correlations of theta activity with the upramp regressor in the non-integration compared to the integration condition. Figure 11C shows the topographical distribution of the weights on the electrodes that define the canonical correlate in the theta band. They have a posterior parietal distribution and are negative. This means that theta power in these parietal channels starts near baseline, and then as the trial progresses, theta power decreases away from baseline.

We next asked whether the dynamics of this theta upramp (the most promising candidate for evidence accumulation) also covaried with individual differences in DDM parameters. We found a significant correlation [*r*(45) = 0.44, *p* = 0.0024] between an individual’s drift rate and the slope of the average theta band time course between 500 and 100 ms pre-response for that same person. There was no significant correlation between the level the time course reached at the end of the response interval and the individual’s behaviorally fitted decision threshold [*r*(45) = 0.08, *p* = 0.48].

## 4 Discussion

We have shown that EEG oscillations exhibit dynamics consistent with evidence accumulation in a perceptual decision making task. In addition, the magnitude of the slopes of these potential “neural accumulators” in the 4–9 Hz theta band covaried with individual differences in the drift rates obtained from the behavioral data.

While correlations with accumulator dynamics occur in different frequency bands, previous studies have implicated theta oscillations in various aspects of decision making (e.g., Jacobs et al., 2006; Cavanagh et al., 2010). For example Jacobs et al. (2006) showed an increase in parietal theta oscillations with decision confidence during a recognition memory task. Cavanagh et al. (2010) observed a correlation between post-decision error monitoring and theta oscillations in frontal regions. Our findings add to this body of evidence. One may wonder what is particular about the theta frequency that would make it suitable for a function in decision making. A modeling study by Smerieri et al. (2010) suggests an answer. They showed that in simulated spiking neural networks of two populations of mutually inhibiting neurons, RTs decreased and drift rates increased with increasing theta power. This effect was specific to the theta range because higher frequencies are too fast to modulate the cell’s membrane potential, which acts as a low-pass filter.

Nevertheless, the correlations of accumulator dynamics with other frequency bands are also not too surprising. For example, a previous study has associated 14–28 Hz beta, rather than theta, oscillations with evidence accumulation (Donner et al., 2009). One reason for that difference with our findings may be that that study was conducted with magnetoencephalography (MEG) rather than EEG recordings. MEG may be better able to detect higher frequency oscillatory activity because of its higher spatial sensitivity (e.g., Dominguez et al., 2005). Another difference with Donner’s study is that the beta oscillations they observed were lateralized, that is, they increased in one hemisphere for one choice and in the other hemisphere for the other choice. While we have examined such lateralized oscillations as well with our model, this did not yield very strong correlations, because the lateralized beta and gamma oscillations only started to rise just before the response.

To further explore how analysis methods affect our results, we redid the same analysis on a Laplace-transformed version of our data, which is a method that improves spatial resolution for some sources, but decreases resolution for other sources (Hauk et al., 2002). This version of the analysis (available from the first author on request) still yielded correlations of EEG with evidence accumulation dynamics, but now the dots-selective accumulator dynamics shifted to more lateral locations and to the alpha and beta bands, quite similar to those observed by Donner et al. (2009). We should also note that our use of wavelets biases us to finding results in the lower frequencies, while multitapers would instead cause a bias toward finding results in higher frequencies (van Vugt et al., 2007). Together, this suggests that evidence accumulation involves oscillations at different frequencies, and the type of oscillations one observes most prominently depends on the recording and analysis methods.

The ramp regressor not only correlates with theta activity but also loads fairly strongly on EEG activity in the 2–4 Hz delta band. Nevertheless, unlike the theta band, the delta band shows a significant correlation with the ramp regressor in *both* the integration and the non-integration control task (Figures 9A,B). This suggests that while theta may be more specific to evidence accumulation, delta may reflect a more generic “on-task” process that is triggered by the dot stimuli on the screen. The relatively large loading on the alpha regressor may reflect bleeding-in of theta activity because there are individual differences in the ranges of alpha and theta oscillations (Klimesch et al., 1993). It may thus be the case that the 9–14 Hz alpha band contains theta accumulator activity from individuals with a high-frequency theta band.

It is also important to consider alternative explanations for the observed correlation with upramp dynamics. For example, the upramp could alternatively reflect time-on-task, which also increases with RT. Arguing against this interpretation is the fact that the correlate of the upramp in the theta band is much lower for the non-integration control task, in which stimulus-viewing-duration has not changed, but the need for evidence accumulation has mostly disappeared. Alternatively, upramp dynamics could reflect response preparation. While some accounts argue that motor preparation already starts at the time of stimulus presentation (Miller and Hackley, 1992), it mostly occurs a few hundred milliseconds before the response (see also Figure 3). A third alternative explanation is that pre-response increases in medial frontal theta increases have previously been associated with response conflict (Cohen and Cavanagh, 2011). The dots and arrows condition do not only differ in accumulation dynamics, but also in the amount of response conflict, and while the effect we observe has different sign and topography from the effects reported by Cohen and Cavanagh (2011), we cannot exclude the possibility that our results represent response conflict.

Another issue to consider is that while the correlation with the ramp regressor is stronger in the theta band than in other frequency bands, evidence accumulation is also a broad-band phenomenon: it is significantly different from zero in almost all frequency bands according to our random effects analysis. Several recent studies have suggested that broad-band increases in oscillatory power reflect increased neuronal spiking more than increases in power in specific frequency bands (Manning et al., 2009). Furthermore, changes in broad-band power have been associated with cognitive processes, such as verbal and spatial memory (Ekstrom et al., 2007; Sederberg et al., 2007).

Although the correlations we obtained between the regressors and the EEG data are on the order of magnitude of correlations obtained from GLMs applied to fMRI data, there is room for methodological improvement. Correlations of fMRI and EEG with task conditions or cognitive models tend to be fairly low due to the large amount of noise in neural data. Nevertheless, we specifically showed that the correlations we obtained are larger than correlations found based on random regressors with a similar temporal structure (Figure 9). Future studies should investigate whether correlations could be improved by applying e.g., Independent Component Analysis (Delorme et al., 2001). In addition, the use of regularization, which zooms in on the informative features in the data, could potentially help to increase the correlation between model dynamics and EEG data.

What may seem surprising about the neural correlate of evidence accumulation in the theta band is that instead of increasing, oscillatory power decreases over the course of the decision interval (Figure 11C). Nevertheless, these decreases in oscillatory power may in fact reflect increases in functional brain activity. This is consistent with (Lorist et al., 2009), who found that oscillatory power increases with fatigue, thereby implying it should decrease with productive task performance. It may also be the case that over the course of evidence accumulation, one moves from a more global mode of processing, in which information is combined from a large number of neurons, to combining information from a much smaller set of neurons associated with less synchronization and lower oscillatory power (von Stein and Sarnthein, 2000). Both of these hypotheses could be tested with more localized neural recordings obtained from e.g., intracranial EEG. A third possibility is that theta may reflect the amount of uncertainty or an urgency to respond (Cisek et al., 2009), rather than the evidence accumulation process *per se*, and that other oscillations (e.g., beta which is more prominently observed in Laplace-transformed EEG and MEG data) may reflect evidence accumulation itself.

Our findings have several implications for future research. First, the correlates of the DDM that are observable in EEG can be used to assess the effect of task manipulations (such as speed-accuracy trade-off or reward rate) on accumulation dynamics. Second, there are large individual differences in decision making (e.g., Forstmann et al., 2010). EEG signatures of neural accumulators may allow us to distinguish different types of participants or strategies, given that individual differences in DDM parameters covaried with the slope of the neural accumulation signal. The “neural accumulators” could thereby soak up some portion of the noise in the model. These “neural accumulators” may also capture individual trial noise, such as attentional fluctuations, although that remains to be proven. Third, we could use the same multivariate methods to clarify the topographical location of possible neural accumulators with fMRI data, which has poorer temporal but better spatial resolution than EEG. Using identical methods for the analysis of EEG and fMRI data in the same task could thus provide new perspectives on data fusion.

Finally, it is important to consider what implications our results have for models of decision making. For example, the non-linearity of the accumulator time courses suggests that evidence accumulation may better be described by a competitive evidence integration than by a linear ballistic accumulator (Brown and Heathcote, 2008). Yet, it is difficult to distinguish between the remaining accumulator models based solely on their dynamics in two-alternative forced choice tasks (Ditterich, 2010). In fact, we have tried different versions of our evidence accumulation model, such as a version where only onset of evidence accumulation changed rather than both onset of evidence accumulation and the slope. We found no appreciable change in our results. However, it is possible to distinguish between some models by employing brief pulses of strong evidence, as in Wong and Huk (2008) and Zhou et al. (2009).

In short, we have developed a novel method for detecting and examining the electrophysiological correlates of model dynamics. Using this method, we have provided evidence for a neural correlate of the dynamics of evidence accumulation in decision making measured in human EEG. Accumulation dynamics were captured best by 4–9 Hz theta oscillations in a set of superior parietal channels, and they covaried with individual differences in DDM parameters fitted to behavioral data.

## Conflict of Interest Statement

The authors declare that the research was conducted in the absence of any commercial or financial relationships that could be construed as a potential conflict of interest.

## Footnotes

^1^http://code.google.com/p/dotsx/

^2^http://pcl.missouri.edu/bf-one-sample
